# Artificial Intelligence's Impact on Drug Discovery and Development From Bench to Bedside

**DOI:** 10.7759/cureus.47486

**Published:** 2023-10-22

**Authors:** K S Vidhya, Ayesha Sultana, Naveen Kumar M, Harish Rangareddy

**Affiliations:** 1 Bioinformatics, University of Visvesvaraya College of Engineering, Bangalore, IND; 2 Pathology, St. George's University School of Medicine, St. George's, GRD; 3 Pharmacology, Haveri Institute of Medical Sciences, Haveri, IND; 4 Biochemistry, Haveri Institute of Medical Sciences, Haveri, IND

**Keywords:** drug discovery research, future of healthcare, nano technology, drug design, artificial intelligence in healthcare

## Abstract

Artificial intelligence (AI) techniques have the potential to revolutionize drug release modeling, optimize therapy for personalized medicine, and minimize side effects. By applying AI algorithms, researchers can predict drug release profiles, incorporate patient-specific factors, and optimize dosage regimens to achieve tailored and effective therapies. This AI-based approach has the potential to improve treatment outcomes, enhance patient satisfaction, and advance the field of pharmaceutical sciences. International collaborations and professional organizations play vital roles in establishing guidelines and best practices for data collection and sharing. Open data initiatives can enhance transparency and scientific progress, facilitating algorithm validation.

## Introduction and background

**Figure 1:**
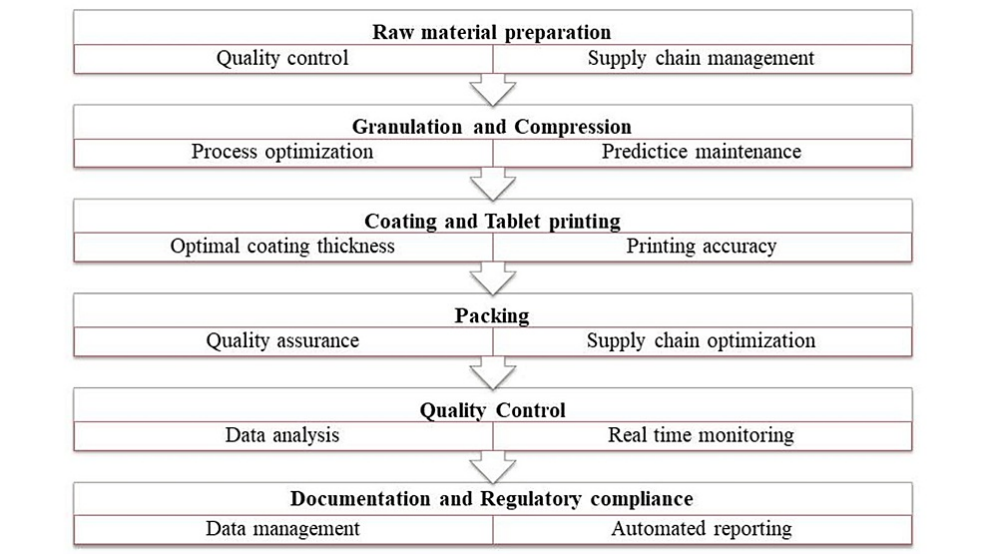
Flowchart showing pharmaceutical drug manufacturing process

The pharmaceutical production process involves the transformation of materials through various stages, progressing from raw materials to active pharmaceutical ingredients, formulation, and ultimately the desired drug product as shown in Figure 1.

**Figure 2:**
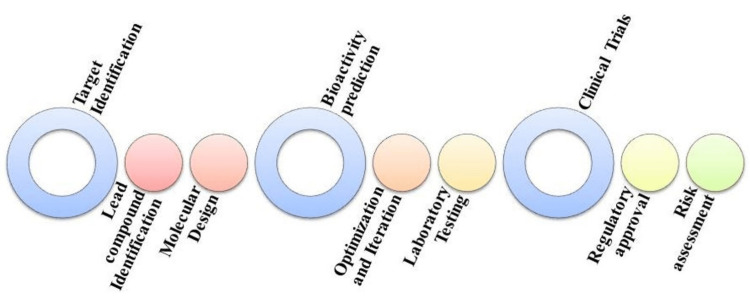
Flowchart showing areas of artificial intelligence application in drug design

Different production routes are often available for the same product. Traditionally, drug development and manufacturing processes have been time-consuming and risky. Nevertheless, recent global pandemics and the integration of artificial intelligence tools, including chatbots, advanced communication methods, and high-speed algorithms, have prompted the industry to transition to a more streamlined and effective development approach. The field of drug delivery systems has witnessed remarkable advancements over the years, enabling targeted and efficient administration of therapeutic agents. Figure 2 depicts the role of artificial intelligence (AI) in the drug designing process. In recent times, the integration of AI has emerged as a transformative force, revolutionizing drug delivery processes [1].

This narrative review aims to explore the role of AI in drug delivery systems, highlighting its applications, benefits, challenges, and future prospects.

## Review

AI-driven formulation design

Utilizing Large Datasets to Identify Correlations and Patterns in Drug Formulations

One of the significant advantages of integrating AI in drug delivery systems is its ability to process and analyze vast amounts of data. By leveraging large datasets, AI algorithms can identify correlations and patterns in drug formulations that may not be apparent through traditional methods [2]. This data-driven approach enables researchers to gain insights into the relationships between various formulation components, their concentrations, and their impact on drug delivery performance [3]. AI algorithms can analyze diverse data sources, including chemical structures, physicochemical properties, and biological activity data, to uncover hidden relationships. By identifying patterns and trends, AI can assist in optimizing drug delivery systems for enhanced efficacy, safety, and stability [4].

Predicting Drug Stability, Solubility, and Release Kinetics Using Machine Learning Algorithms

Drug stability, solubility, and release kinetics are critical factors influencing the efficacy and performance of drug delivery systems [5]. AI-powered machine learning algorithms can predict these properties by learning from available experimental and computational data. By analyzing the relationships between molecular structures, formulation components, and environmental factors, AI algorithms can generate accurate predictions [6]. Machine learning models can capture complex relationships and non-linear dependencies, allowing for the prediction of drug stability under various storage conditions, the solubility of drugs in different formulations, and the release kinetics from different delivery systems [5]. This information can guide formulation design, ensuring optimal drug delivery and therapeutic outcomes.

Accelerating the Formulation Development Process Through Virtual Screening and Optimization

Traditionally, the formulation development process involves extensive experimentation and iterative testing, which can be time-consuming and costly. AI offers the potential to expedite this process through virtual screening and optimization techniques.

**Figure 3:**
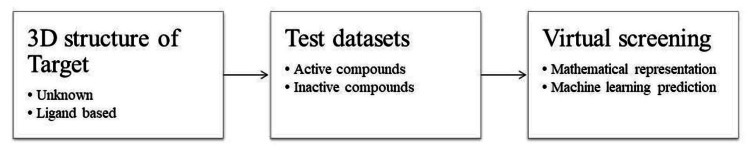
Flowchart of overview of Virtual Screening

Virtual screening involves the rapid screening of large compound libraries to identify potential candidates for formulation development. By leveraging AI algorithms, researchers can analyze molecular properties, structure-activity relationships, and other relevant parameters to prioritize compounds with desirable characteristics as shown in Figure 3.

AI-guided optimization techniques further enhance the formulation development process by exploring vast formulation design spaces. By applying optimization algorithms, AI can identify optimal combinations of formulation components, concentrations, and manufacturing processes to achieve desired drug delivery properties [7].

Enhancing Drug Delivery System Performance by Leveraging AI-Guided Formulation Design

AI can significantly improve drug delivery system performance by guiding formulation design. By analyzing data on the physicochemical properties of drugs and excipients, as well as their interactions, AI algorithms can recommend optimal formulation strategies. For example, AI can suggest suitable excipients or carriers that improve drug solubility, stability, or release profiles [8]. It can also identify innovative drug delivery approaches, such as nanoformulations or lipid-based systems, which enhance drug targeting and tissue penetration [9].

Additionally, AI can account for individual patient factors, such as demographics, genetics, and disease characteristics, to enable personalized drug delivery. This allows for tailored formulations that optimize therapeutic efficacy while minimizing side effects. By leveraging AI-guided formulation design, drug delivery systems can be optimized for specific drugs and patient populations, leading to improved treatment outcomes and enhanced patient care [10].

Overall, the integration of AI in drug delivery systems offers immense potential for advancing formulation design, predicting drug properties, and optimizing delivery performance. By harnessing the power of AI and large datasets, researchers can accelerate the development of safe and effective drug delivery systems, ultimately improving patient outcomes and advancing the field of pharmaceutical sciences.

Intelligent Nanocarriers

“Nanocarriers are colloidal drug carrier systems having submicron particle size typically <500 nm” [11]. AI methodologies are consistently emerging as solutions for diverse challenges within nanotechnology. These encompass the development of nanosystems, nanocomputing, and the integration of AI techniques into the formulation of nanoscale simulation methodologies. This emphasis is placed on enhancing computational efficiency, optimizing parameter estimation, predictive modeling, and facilitating system simulation. In recent times, artificial intelligence methods have been harnessed for the design, characterization, and fabrication of drug delivery nanosystems aimed at refining drug administration. A significant portion of AI techniques is directed towards the analysis and interpretation of genetic and biological data. This cohesive approach has significantly expedited the drug discovery process, enabling the identification of diverse attributes of small molecules and enhancing predictive capabilities [12]. For instance, multiple AI-driven methodologies are being proposed for the prediction of drug combination effectiveness through synergies between drugs. In this context, potential avenues are being explored for AI-guided optimization in combination drug delivery, utilizing various classes of nanoparticles to enhance drug localization at tumor sites [13]. Designing and optimizing nanocarriers for targeted drug delivery is a promising area in drug delivery research, and AI algorithms can play a crucial role in enhancing their performance.

Designing and Optimizing Nanocarriers for Targeted Drug Delivery

**Figure 4:**
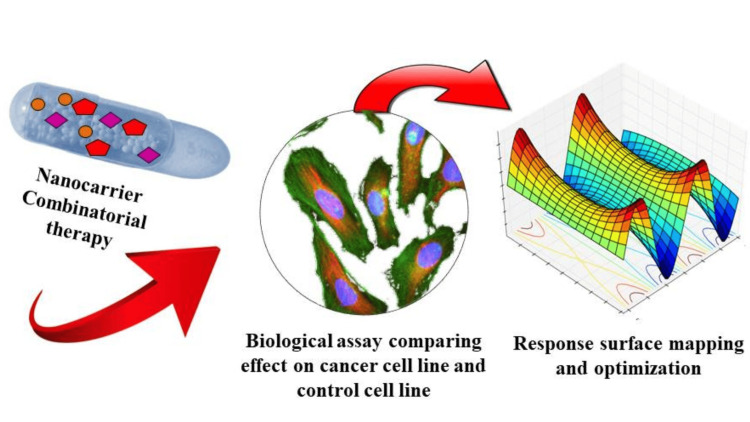
Schematic representation of Feedback System Control (FSC) in optimizing nanocarrier combination therapy This file is licensed under the Creative Commons Attribution-Share Alike 4.0 International license.

Nanocarriers, such as liposomes, nanoparticles, and polymeric micelles, have emerged as effective platforms for delivering therapeutic agents to specific target sites [14,15]. AI algorithms can aid in the design and optimization of nanocarriers by analyzing complex relationships between their properties and performance. By utilizing AI algorithms, researchers can generate predictive models that consider various parameters, including the physicochemical properties of nanocarriers, drug characteristics, and biological factors [15]. These models can guide the selection of appropriate nanocarrier materials, size, surface modifications, and other formulation parameters for optimal drug delivery. AI-based Feedback System Control (FSC) platform is used to optimize combinatorial drug design especially in cancer chemotherapy with nanocarriers as shown in Figure 4 [16].

The FSC platform is employed to construct phenotypic response surfaces by establishing correlations between drugs and their respective therapeutic windows. These windows are determined based on variations in viability assays between cancer and control cell lines, offering insights into both effectiveness and toxicity. Utilizing this approach, FSC effectively identifies comprehensive combinatorial treatment regimens for individual cancer cell lines, guided by the calibrated phenotypic response surface that determines the globally optimized combination of the complexes [16].

Enhancing Encapsulation Efficiency and Drug Release Profiles

Encapsulation efficiency refers to the amount of drug loaded within a nanocarrier, and it significantly influences the therapeutic efficacy of the drug delivery system [17]. AI algorithms can assist in improving encapsulation efficiency by analyzing data on the interactions between drugs and nanocarrier components. AI and machine learning techniques may be employed for the analysis of data gathered during the research and systematic review studies. Through the application of various algorithms, exploratory data analysis (EDA) is conducted to develop predictive models. These models aim to support future researchers in achieving highly accurate and reproducible results. AI algorithms can identify key factors affecting drug loading and release. This includes the optimization of formulation parameters such as polymer-drug ratios, encapsulation techniques, and drug loading protocols. By predicting and modeling drug-nanocarrier interactions, AI algorithms can guide the formulation design process to achieve higher encapsulation efficiency and control drug release kinetics [18].

Intelligent Control of Nanocarrier Surface Properties for Improved Cellular Targeting and Drug Delivery

The surface characteristics of nanocarriers play a pivotal role in determining their bioavailability, stability, cellular absorption, and distribution within the body. The zeta (ζ) potential, which signifies the surface charge, provides insights into potential electrostatic interactions among nanocarriers, influences their tendency to aggregate, and aids in choosing suitable coating materials [19].

AI enables the analysis and prediction of surface modifications' effects, like ligand conjugation or coating with targeting agents, on nanocarrier-cell interactions. It identifies optimal ligands or strategies based on molecular and cellular data. The aim of incorporating automation and AI is to improve the customization of targeted therapeutic nanocarriers for distinct cell types and individual patients [20].

Molecular modeling analyses of drug delivery systems involving nanocarriers have predominantly concentrated on (i) assessing nanocarrier structure and arrangement, (ii) evaluating nanocarrier transport and interactions, (iii) appraising nanocarrier surface characteristics, and (iv) examining nanocarrier binding to diverse surfaces [21].

Additionally, AI aids in designing stimuli-responsive nanocarriers for triggered drug release in response to pH, temperature, or enzyme changes. Optimization algorithms identify effective components and control mechanisms. Integrating AI enhances nanocarriers' surface properties, improving targeting, internalization, and drug release, thus enhancing drug delivery efficiency. Polymeric nanocarriers can be tailored as stimuli-responsive systems that trigger drug or gene release in response to specific stimuli, either originating within the body viz., pH, enzymes, temperature, redox values, hypoxia, glucose levels or externally applied such as light, magnetism, ultrasound, electrical pulses [22].

AI-enabled drug release systems

Developing smart drug delivery systems that can respond to physiological cues and adapt to patient-specific needs is an exciting frontier in the field of drug delivery. AI algorithms play a crucial role in monitoring, modulating, and optimizing drug release in real-time, enabling precise and personalized drug administration through closed-loop feedback systems [23].

Smart Drug Delivery Systems Responding to Physiological Cues

**Figure 5:**
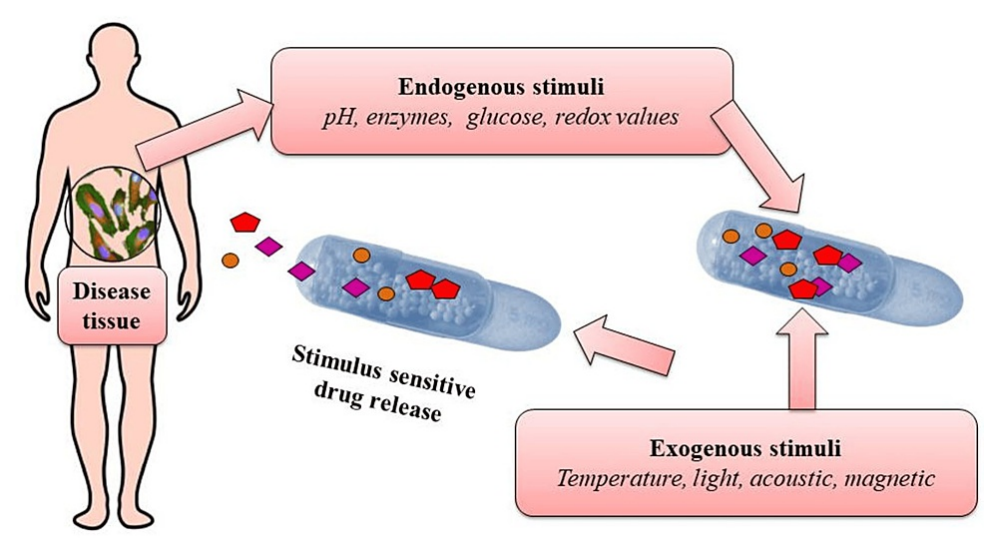
Stimulus responsive nanoplatforms in smart drug delivery system This file is licensed under the Creative Commons Attribution-Share Alike 4.0 International license.

Smart drug delivery systems are designed to sense and respond to specific physiological cues or disease conditions in the body. AI algorithms can analyze real-time data from sensors, biomarkers, or imaging techniques to detect changes in the target site or patient's physiological state. Smart drug delivery systems refer to nanoplatforms containing drug payloads that prevent indiscriminate drug release in the bloodstream, releasing them exclusively at targeted sites achieved through active or passive targeting strategies. These meticulously engineered smart or stimuli-responsive nanoplatforms can react to internal or external stimuli, as depicted in Figure 5 [23].

Internal triggers encompass pH fluctuations, hormone levels, enzyme concentrations, various bio-molecules, glucose levels, and redox gradients, all of which relate to the specific pathological characteristics of a disease. Conversely, external triggers such as temperature, magnetic fields, ultrasound, light, and electric pulses or high-energy radiation can also be harnessed to initiate or enhance drug release at the affected regions [23]. For example, in cancer therapy, AI algorithms can analyze data related to tumor size, metabolic activity, or biomarkers to assess the disease status. Based on this information, the drug delivery system can respond by modulating drug release, adjusting dosage, or activating specific drug delivery mechanisms to target the tumor more effectively [23].

Utilizing AI Algorithms to Monitor and Modulate Drug Release

AI algorithms can enable real-time monitoring and control of drug release from the delivery system. By analyzing patient-specific data, such as drug levels in the bloodstream, physiological parameters, or biomarkers, AI algorithms can make informed decisions regarding drug dosage, timing, or release rates [24]. These algorithms can continuously analyze the collected data and adjust the drug release profile accordingly. By integrating AI with sensors, biosensors, or wearable devices, drug delivery systems can provide personalized and adaptive therapy, optimizing therapeutic outcomes [24].

Closed-Loop Feedback Systems for Precise, Personalized Drug Administration

Closed-loop feedback systems combine real-time data monitoring with AI algorithms to achieve precise and personalized drug administration. These systems create a feedback loop between the drug delivery system, the patient, and the AI algorithms [25].

The process begins with continuous data collection from sensors or wearable devices monitoring relevant physiological parameters or drug levels [26]. "Wearables" encompass a wide range of devices, including smart watches, electronic contact lenses, wearable tattoos, smart patches, electronic textiles, exosuits, electronic footwear, prosthetic devices, subcutaneous sensors, and more. These devices are utilized to measure electrophysiological or biochemical signals and, more recently, have been employed for point-of-care drug monitoring. Wearable technology has also been applied in areas such as gait training, motion detection, human activity recognition, robotics, and various human-machine interfaces [27].

**Figure 6:**
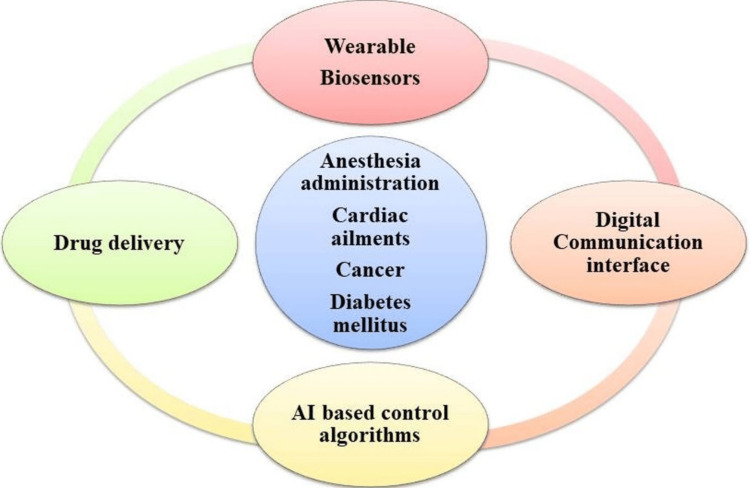
Integrating wearable technologies with drug delivery systems This file is licensed under the Creative Commons Attribution-Share Alike 4.0 International license.

AI algorithms analyze this data to assess the patient's condition and therapeutic needs, making decisions about drug release or dosage adjustments. The drug delivery system, equipped with actuators for controlled release, then adjusts drug release according to AI instructions. This closed-loop system tailors drug delivery in real-time, adapting to disease progression or physiological changes. Incorporating closed-loop feedback systems into drug delivery, AI enables precise therapy adjustments, maximizing efficacy and minimizing side effects. This personalized approach has potential to enhance treatment outcomes, patient comfort, and therapeutic interventions, offering real-time monitoring, modulation, and optimization of drug release based on physiological cues and patient-specific needs [28]. Figure 6 addresses the potential settings where wearables can enhance healthcare delivery, the flow of data from wearables to inform health decisions, the diverse healthcare areas that current wearables can impact (some with robust evidence, while others are emerging), the challenges and restrictions in adopting wearable tech in healthcare, and the concept of a unified platform that integrates data collection from wearable devices, analytics, and the delivery of interventions to create a comprehensive healthcare monitoring system [28].

Predictive drug release modeling

AI techniques can model and predict drug release profiles from different delivery systems by analyzing data on formulation parameters, drug properties, and system characteristics. Machine learning algorithms enable the formulation of complex relationships between these factors, optimizing drug delivery systems for desired outcomes [5]. AI can also personalize drug release by analyzing patient-specific data like genetics and medical history, allowing tailored delivery systems to match individual needs, enhancing treatment efficacy, and minimizing side effects [6].

Various machine learning algorithms, such as neural networks, support vector machines, and random forests, have been applied to drug release modeling. These algorithms excel at recognizing intricate patterns within datasets, enabling accurate predictions of release kinetics. AI models integrate diverse data sources, including physicochemical properties of drugs, formulation characteristics, and in vitro and in vivo data. This holistic approach allows for a comprehensive understanding of drug release dynamics [29].

The drug release optimization process, crucial for meeting formulator requirements, typically involves repetitive testing and batch preparation, leading to significant tedium and time consumption [30]. Incorporating AI into drug formulation streamlines this process by predicting release patterns, reducing the number of necessary optimization runs, and consequently, cutting down both labor and production costs. AI can effectively anticipate drug release, dissolution profiles, and disintegration times, facilitating the selection of the optimal batch for subsequent scaling. Researchers have successfully utilized AI algorithms, including artificial neural networks (ANNs), support vector machines (SVM), and regression analysis, to predict dissolution profiles in hydrophilic matrix-type sustained-release tablets. Data for modeling drug release were gathered using process analytical technology (PAT) and critical material attributes. Among these attributes, particle size distribution emerged as the most critical variable in predictive modeling. The evaluation metrics identified the most accurate models through ANN implementation [31,32].

Enhancing Dosage Regimen Optimization and Minimizing Side Effects Through AI-Based Modeling

AI-based modeling can contribute to the optimization of dosage regimens and the minimization of side effects. By integrating patient-specific data, clinical parameters, and drug release profiles, AI algorithms can generate computational models that simulate the pharmacokinetics and pharmacodynamics of a drug [33]. These models can consider various factors, such as drug concentration, dosing frequency, and individual patient characteristics, to predict how different dosage regimens will impact drug efficacy and safety. By leveraging AI algorithms, researchers can optimize dosing schedules to maximize therapeutic benefits and minimize adverse effects. Moreover, AI can assist in identifying potential side effects or drug interactions by analyzing large datasets and detecting patterns that may not be easily discernible through traditional methods. This information can guide clinicians in making informed decisions regarding dosage adjustments or medication combinations to enhance patient safety [34].

The speed at which a drug dissolves in a biological fluid, known as its dissolution rate, plays a pivotal role in determining how effectively it can be absorbed and deliver therapeutic effects. AI has made substantial strides in forecasting these dissolution rates, thus aiding in refining drug formulations and dosages. By sifting through extensive experimental data, AI models can pinpoint critical physicochemical traits and molecular attributes that impact the dissolution process. These models harness machine learning algorithms to grasp intricate patterns and connections between drug properties and dissolution rates, resulting in precise forecasts. AI provides insights into how various drug formulations behave during dissolution, enabling the development of more efficient drug delivery systems and assisting in the selection of ideal formulation approaches to enhance drug solubility and absorption. This progress in dissolution rate prediction, empowered by AI, equips pharmaceutical researchers with valuable instruments to expedite drug development, optimize formulation tactics, and ultimately enhance patient outcomes [35].

Incorporating Multi-Omics Data and Network Analysis to Improve Target Identification

The integration of multi-omics data and AI-driven network analysis enhances the identification of disease targets for drug delivery. Multi-omics data, encompassing genomics, transcriptomics, proteomics, and metabolomics, offers a comprehensive view of disease processes and molecular interactions. AI algorithms analyze this data to construct molecular networks, identifying key disease-associated targets and pathways while considering their interconnected nature, thus aiding in the prioritization of drug delivery targets. Additionally, AI algorithms delve into extensive biological networks, such as protein-protein interaction or gene regulatory networks, to uncover potential drug targets and pathways often overlooked by traditional methods. This network analysis, coupled with AI techniques, provides a systems-level understanding of disease processes, improving target identification for drug delivery. These AI-driven tools enable researchers to analyze multi-omics data, conduct network analysis, and develop precise targeting strategies, ultimately enhancing drug delivery efficacy and advancing precision medicine, promising improved therapeutic outcomes [36].

Challenges and future perspectives

Ethical and Regulatory Considerations

The ethical implications of AI-driven drug delivery systems are crucial considerations that need to be addressed to ensure the safe and responsible implementation of these technologies. Here are some key aspects to consider:

Transparency, Fairness, and Accountability in Decision-Making Algorithms

AI algorithms used in drug delivery systems should be transparent, meaning the decision-making processes and underlying criteria should be explainable and understandable. Transparency ensures that clinicians, regulators, and patients can comprehend how the algorithms arrive at their conclusions, enhancing trust and accountability. Fairness is another critical aspect. AI algorithms should be designed and trained in a way that avoids bias and discrimination, ensuring equitable access to drug delivery systems and therapies. The algorithms should not perpetuate or amplify existing healthcare disparities based on factors such as age, gender, race, or socioeconomic status. Accountability is necessary to ensure that responsibility for the decisions made by AI-driven drug delivery systems can be assigned and addressed appropriately. Clear guidelines and mechanisms should be established to determine liability in cases of system errors or adverse events [37].

Regulatory Frameworks for Safe and Responsible Implementation

Regulatory frameworks play a vital role in ensuring the safe and responsible implementation of AI in drug delivery systems. These frameworks should be designed to evaluate and approve AI algorithms, considering factors such as safety, efficacy, and ethical considerations. A significant step in addressing the regulatory and ethical considerations of AI in drug development is the U.S. Food and Drug Administration's (FDA) release of a discussion paper titled "Utilizing Artificial Intelligence & Machine Learning in Drug and Biological Product Development." This document offers insights into AI's role in drug discovery, nonclinical research, and clinical research, accompanied by recommended best practices for AI and machine learning application. The FDA's initiative represents a significant milestone in overseeing AI's use in healthcare, opening doors to fresh opportunities in the sector. It signifies the acknowledgment of both the potential advantages and challenges tied to AI in drug development, laying the groundwork for future regulatory advancements in this field [38].

Informed Consent and Patient Autonomy

In AI-driven drug delivery systems, ensuring informed consent is a paramount ethical concern. Patients should receive clear, comprehensible information about AI algorithms' involvement in their treatment and its potential impact on their care. This empowers patients to make informed decisions regarding treatment options, understanding the advantages, risks, and limitations of AI-driven drug delivery systems. Effective communication by healthcare professionals, explaining AI's role and addressing patient queries, is vital in facilitating patient autonomy in decision-making [39].

Standardized Data Collection and Sharing Practices

Establishing standardized data collection and sharing practices is essential for consistency, comparability, and reproducibility in AI-driven drug delivery research. This entails defining common data elements, protocols, and metadata standards to consistently capture relevant information across studies and healthcare settings. International collaborations and professional organizations can guide these efforts by setting data collection guidelines. Encouraging adherence to these standards fosters a culture of data sharing, benefiting the scientific community. Supporting open data initiatives, when ethically viable, enhances transparency. Providing access to anonymized datasets and promoting data publication with research findings aids scientific progress and validates AI algorithms. Overcoming data-related challenges requires collaborative, standardized, and ethical approaches, enabling the field of AI-driven drug delivery to leverage diverse, reliable datasets for robust algorithm development and advancing personalized medicine [40].

Collaboration and interdisciplinary research

Promoting collaboration among researchers, clinicians, engineers, and data scientists is essential for advancing AI-driven drug delivery systems. This multidisciplinary approach combines domain knowledge, technical skills, and clinical insights to drive innovation and address challenges effectively. Researchers and clinicians bring expertise in drug delivery, pharmacology, and patient care, guiding AI-driven system development and aligning AI algorithms with clinical needs. Engineers and data scientists contribute technical skills in AI, machine learning, data analysis, and algorithm development, enabling the optimization of AI-driven drug delivery systems through joint collaboration. Initiatives like joint research projects, interdisciplinary workshops, and research centers facilitate knowledge exchange among experts, promoting idea sharing, resource pooling, and funding opportunities for multidisciplinary AI-driven drug delivery research. Developing cross-disciplinary education and training programs equips professionals with shared skills and perspectives, enhancing communication and teamwork to foster effective collaboration.

## Conclusions

Artificial intelligence is poised to revolutionize the field of drug delivery systems by enabling more precise, personalized, and efficient therapeutic interventions. The integration of AI in formulation design, drug release systems, targeting strategies, and monitoring techniques holds tremendous potential for improving treatment outcomes and patient care. However, addressing ethical, regulatory, and data-related challenges will be critical to realizing the full potential of AI in drug delivery. With continued research, collaboration, and technological advancements, AI is expected to shape the future of drug delivery systems, ushering in a new era of precision medicine.
